# Light-Programmed Bistate Colloidal Actuation Based on Photothermal Active Plasmonic Substrate

**DOI:** 10.34133/research.0020

**Published:** 2023-01-10

**Authors:** Fangfang Deng, Juntao Chen, Junxiang Xiang, Yong Li, Yan Qiao, Ze Liu, Tao Ding

**Affiliations:** ^1^ Key Laboratory of Artificial Micro- and Nano-structures of Ministry of Education of China, School of Physics and Technology, Wuhan University, Wuhan 430072, China.; ^2^ Department of Engineering Mechanics, School of Civil Engineering, Wuhan University, Wuhan, Hubei 430072, China.; ^3^ Beijing National Laboratory for Molecular Sciences (BNLMS), Laboratory of Polymer Physics and Chemistry, CAS Research/Education Center for Excellence in Molecular Sciences, Institute of Chemistry, Chinese Academy of Sciences, Beijing 100190, China.

## Abstract

Active particles have been regarded as the key models to mimic and understand the complex systems of nature. Although chemical and field-powered active particles have received wide attentions, light-programmed actuation with long-range interaction and high throughput remains elusive. Here, we utilize photothermal active plasmonic substrate made of porous anodic aluminum oxide filled with Au nanoparticles and poly(*N*-isopropylacrylamide) (PNIPAM) to optically oscillate silica beads with robust reversibility. The thermal gradient generated by the laser beam incurs the phase change of PNIPAM, producing gradient of surface forces and large volume changes within the complex system. The dynamic evolution of phase change and water diffusion in PNIPAM films result in bistate locomotion of silica beads, which can be programmed by modulating the laser beam. This light-programmed bistate colloidal actuation provides promising opportunity to control and mimic the natural complex systems.

## Introduction

Active particles [1], due to their locomotive and adaptive features, have been regarded as ideal models to mimic many biological systems and their collective behaviors such as cellular metabolism, biological clock, fish schooling, population fluctuation, etc. [2], which are largely controlled by signal molecules, cascade reactions, and quorum sensing [3]. Mimicking such oscillatory motion not only deepens the understanding of the dynamic and adaptive self-assembly process [4] but also offers the opportunity of developing new types of nanomachines and nanotransducers [5–7]. Autonomous colloidal oscillation has been applied to mimic such oscillatory movements, which is driven by chemical oscillation [8,9] or DNA hybridization [10]. However, such autonomous oscillation is usually slow and difficult to control [11,12]. Thus, advanced colloidal manipulation requires on-demand controllability and directionality, which is substantial for digital and optical applications [13,14]. Although different manipulation mechanisms such as hydrodynamics [15], (opto)thermal gradient [16–21], photoelectric force [22,23], optoelasticity [24,25], and osmosis diffusion [26–28] are developed, substantial Brownian noises persist because of the dispersive nature of the system, which makes their manipulation less precise and sometimes unstable. Moreover, bistate switching control remains a big challenge though it is fundamental for colloidal computation [29].

Actuation driven by optical tweezers is remotely controllable and on-demand [30], which is a fascinating technique to manipulate colloidal particles for both fundamental understanding [31] and intriguing applications [32]. The phototaxis of colloidal analog provides an ideal model for the understanding of the physical mechanism of many biological behaviors [33]. It has recently been proposed that with suppressed Brownian motion on solid substrate, optical manipulation can generate diverse configurations of nanoassemblies [34–36]. However, because of the limited interaction range of optical force, both the amplitude of locomotion and the capacity of control are small, which leads to less efficiency in particle manipulation [37,38].

Surface force induced by surface tension and elastic force caused by volume change are much larger compared to optical force, which appear an efficient tool for droplet manipulation [39–41]. It would be a reliable and delicate means to control the motion of nano-/micro-objects with high throughput and long interaction range, but seldom has it been applied for bistate colloidal actuation largely due to the irreversibility of the manipulation [42–45].

Here, we devise a hybrid plasmonic surface with large surface force and elastic force, which is induced by the coupled photothermal effect and water diffusion in poly(*N*-isopropylacrylamide) (PNIPAM) so that colloidal particles attached to the surface can be actuated with light on and switch back with light off, making them optically programmed oscillators. This hybrid plasmonic substrate is made of anodic aluminum oxide (AAO) template filled with Au@PNIPAM core–shell nanoparticles (NPs). The surface of the substrate was overcoated with an extra thin layer of PNIPAM before depositing silica beads on top. The plasmonic Au NPs within the AAO template functionalize as the heating source under continuous wave (CW) laser illumination, which rises the local temperature above the phase transition temperature (*T*_c_) of PNIPAM. As a result, the thermal gradient near the edge of phase change region produces surface force, and the heat-induced phase change results in elastic force, which drives the motion of silica beads back and forth within several micrometers, depending on the thickness of PNIPAM film. Such neat design of hybrid plasmonic surfaces not only shows how phase-change-induced forces can be used for colloidal manipulation but also provides unique opportunity and implications for biomimicking and cellular mechanics.

## Results

**Fig. 1.**
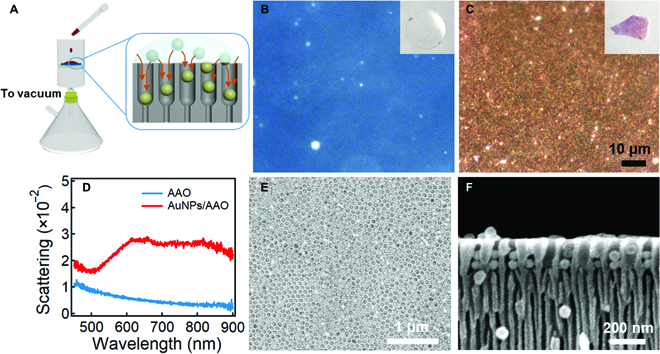
Hydrodynamic self-assembly of Au NPs in AAO template. (A) Experimental setup of hydrodynamic self-assembly of Au NPs in AAO template. DF images of AAO template (B) before and (C) after vacuum filtration. Insets are the images of corresponding samples. (D) DF scattering spectra of AAO template before and after filled with Au NPs. SEM images of Au NPs filled in the AAO template (E) top view (F) cross-sectional view. Note that a few channels without Au NPs are unintentionally introduced during the sample preparation.

Hydrodynamic force is commonly used to align and assemble molecules/particles for photonic and biological applications [46,47]. However, because of dynamic nature of these self-assemblies, their configuration cannot be preserved unless being fixed by polymers [48]. The key of successful hydrodynamic self-assembly is that the strong repulsion between the colloidal particles should be maintained either electrostatically or sterically to avoid aggregation during the shear flow of the fluid. Thus, we adopt Au@PNIPAM core–shell NPs as the building blocks that are stable and thermally responsive. The PNIPAM coating also facilitates the growth and adhesion of PNIPAM overlayer for thermal active substrate. The Au@PNIPAM core–shell NPs have a shell thickness of 2 nm (Fig. S1), and we apply hydrodynamic flow via vacuum filtering to fill the nanopores (~85 nm) of AAO template with these NPs in a sequential manner (Fig. 1A). The AAO template with the narrower opening end (~20 nm) blocks the Au@PNIPAM NPs from completely going through (inset of Fig. 1A), enabling high filling ratio of the NPs. With both the physical confinement and liquid flow under pressure gradient, nematic colloids can be formed and preserved in the AAO template [49]. The ultrathin AAO template (~20 μm in thickness; Fig. S2) changes from semitransparent to pink after several cycles of filtering (inset of Fig. 1B and C), which appears orange under dark-field (DF) optical microscope (Fig. 1C). The scattering spectra show broad resonance peak from 600 to 800 nm, suggesting the mode coupling between the Au NPs, while for pure AAO template, no plasmon resonances can be found. The filling ratio of the Au NPs in the pores can reach up to 90% (the dark bits are unfilled nanopores; see Fig. 1E), most of which are made of Au dimers (Fig. 1F).

**Fig. 2.**
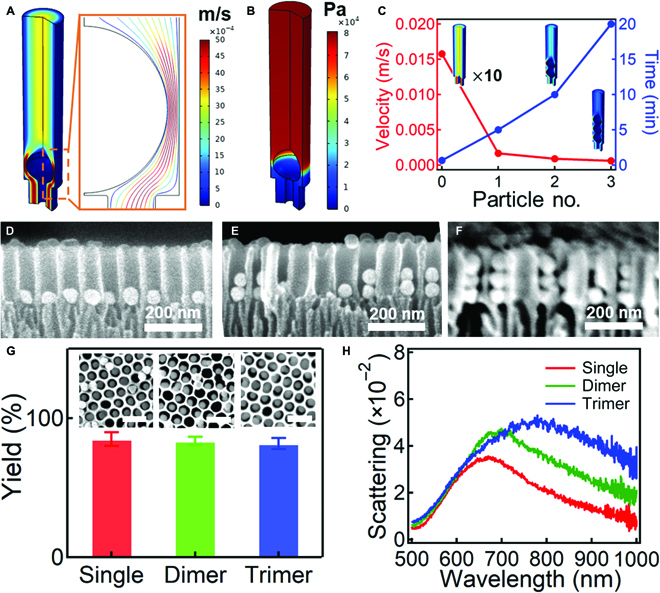
Mechanistic insights on the hydrodynamic self-assembly process within AAO template. Finite-difference time-domain simulations of (A) flow field and (B) pressure field within the AAO channel when one Au NP is filled in the channels. (C) Change of the average flow velocity and the assembly time required with the number of Au NPs in the AAO channels. Insets are the flow velocity profile with presence of 1, 2, and 3 Au NPs. SEM images of Au NPs within the AAO templates, (D) single Au NPs, (E) dimer Au NPs, and (F) trimer Au NPs. (G) Yield of Au NPs in the AAO channels for single, dimer, and trimer particles. Insets are the typical SEM images. Scale bars, 200 nm. (H) DF scattering spectra of single, dimer, and trimer Au NPs within the AAO templates in air.

The hydrodynamic self-assembly process was analyzed via finite element method (Fig. 2A to C). For empty AAO channels, the simulation results suggest that the liquid goes in pores with a speed up to ~15 mm/s. However, when one Au NP has been deposited at the bottom of the channel, the flow speed drops drastically to 5 mm/s (Fig. 2A) with hydrostatic pressure build-up to ~80 kPa (Fig. 2B). For Au trimers, the assembly time takes up to 20 min, as the flow velocity is less than 1 mm/s as indicated by simulation (red line in Fig. 2C). This is reasonable since the filled Au NPs leads to the reduction of effective channel diameter, which greatly reduces the mean flow speed of liquid. As a result, other Au NPs prefer to fill other channels without Au NPs, where the flow speed is much faster. Therefore, the Au NPs prefer to fill the channels in a layer-by-layer manner, which can be controlled by the filtration suction time (Fig. 2C). Experimentally, the yield of single, dimer, and trimer Au NPs is very high with filling ratio of 80% to 90% (Fig. 2G and the insets). Note that the channels without Au NPs observed in the cross-sectional view (Fig. 2D to F) are mainly introduced by intentionally breaking the AAO template as required for clear scanning electron microscopy (SEM) imaging. The single Au NP arrays filled in the AAO pores have a plasmonic resonance around 650 nm (red line in Fig. 2H), which is due to the high refractive index surroundings of the AAO template and weak coupling between the neighboring Au NPs (Fig. 2D). It is also noted that with the increasing number of Au NPs within the channels, the redshift of the plasmon resonances is due to the increased coupling strength of dimers and trimers (green and blue lines in Fig. 2H). The absorption spectra of single, dimer, and trimer Au NPs show similar trend of redshift with increasing number of Au NPs in the channel (Fig. S3) but are overall blueshifted as compared to their scattering spectra (Fig. 2H).

**Fig. 3.**
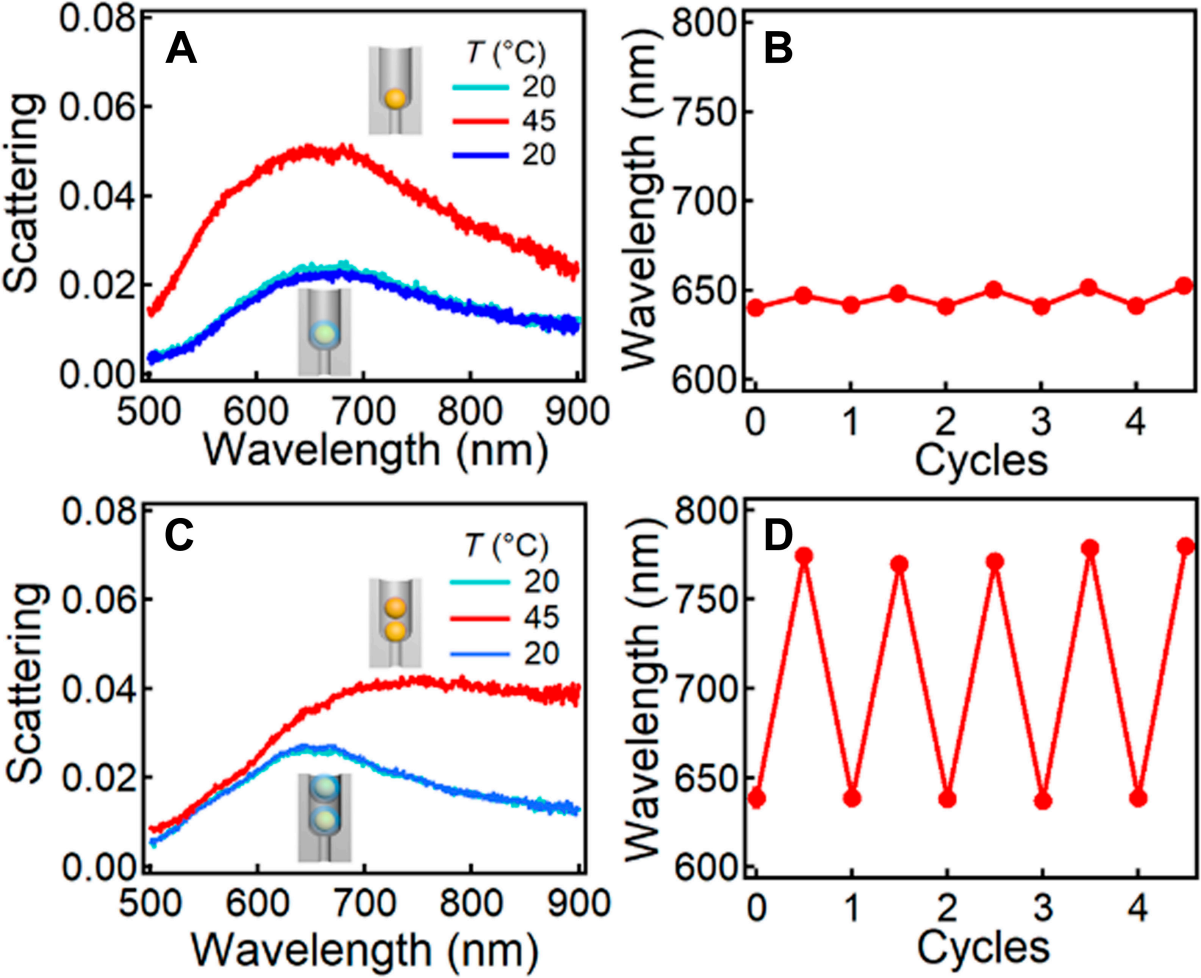
Dynamic actuation of Au NPs within the AAO template via temperature responsive polymers (PNIPAM) in aqueous environment. Scattering spectra of (A) single and (C) dimer Au NPs within AAO template with changing temperature from 20 to 45 °C and back to 20 °C and the corresponding change of resonance wavelength with cycles of heating and cooling. (B) single Au@PNIPAM NPs and (D) dimer Au@PNIPAM NPs. The spectra are fitted with Gaussian to determine their peak positions.

Since the Au NPs are coated with PNIPAM shell, we can actuate the assembled Au NPs within the AAO channels by adjusting the temperature, which shows distinct plasmon resonances for different assembly configurations (Fig. 3). For single Au@PNIPAM NPs within the AAO template, only intensity modulation with slight shift (~5 nm) of plasmon resonances was observed, which is largely due to the increase in refractive index of the PNIPAM shells after phase transition (Fig. 3A) [50]. For dimer Au@PNIPAM NPs, both plasmon intensity and resonance wavelength change dramatically mainly because of the decrease in gap size between the Au NPs (Fig. 3C). In both cases, the reversibility of plasmon shift is robust and the largest shift can go up to ~120 nm (Fig. 3B and D). Note that this thermoresponsive tuning process is performed in aqueous environment with PNIPAM in the nanogaps, which enlarges the separation of the Au dimers at room temperature. Therefore, their scattering peak (blue line in Fig. 3C) is blueshifted as compared to the dried state (green line in Fig. 2H), which also appears similar to the single Au NPs in the AAO channels (blue lines in Fig. 3A).

**Fig. 4.**
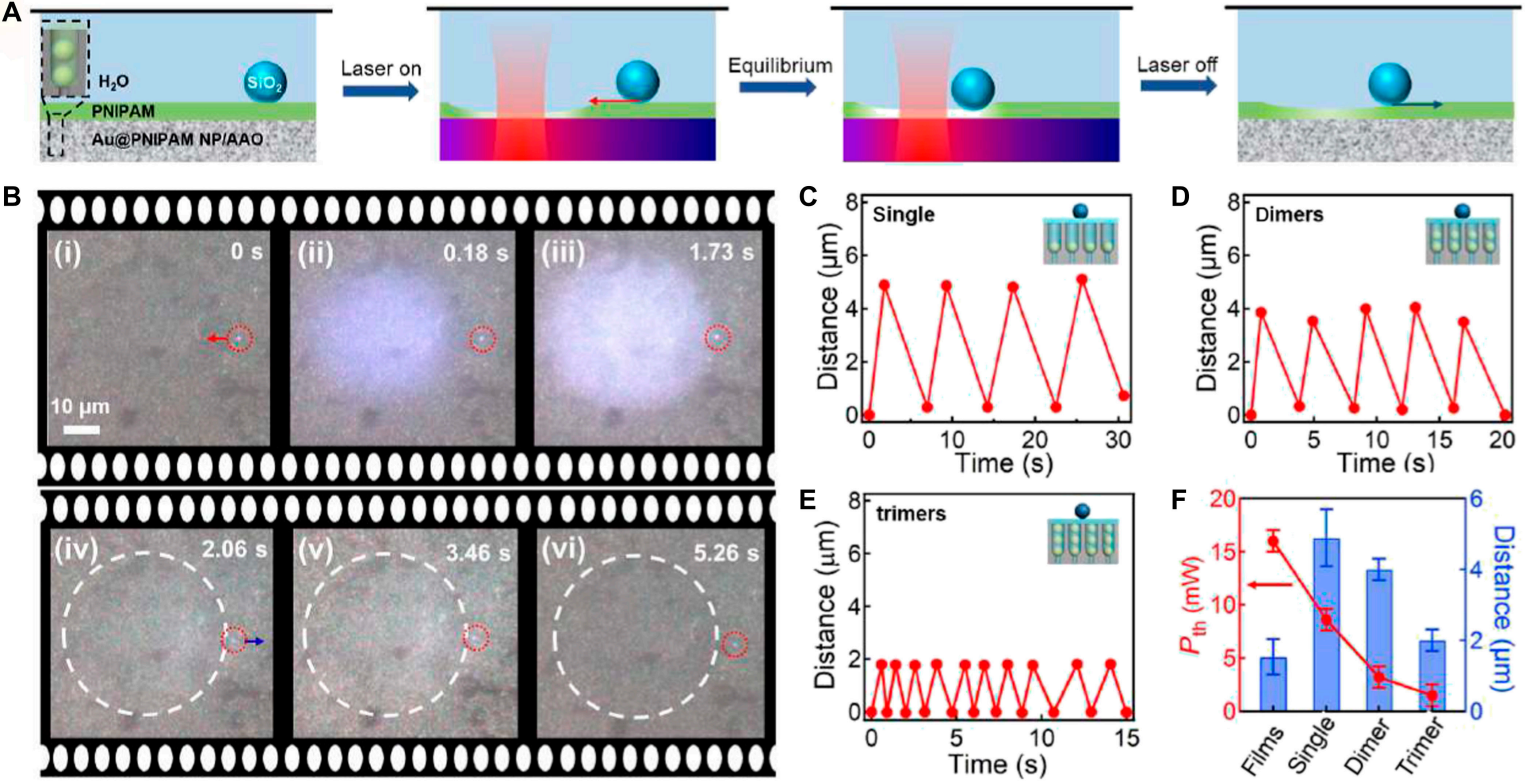
Light-induced oscillation of silica bead on hybrid plasmonic substrate. (A) Schematic of light-induced oscillation of silica bead and (B) its microscopic snapshots of oscillatory movement of silica bead on single Au@PNIPAM NP/AAO substrate with one cycle of laser switched on and off. The laser power applied was 8.6 mW. Laser was switched off in (iv). The white dashed circles in (iv to vi) represent the white phase transition region as shown in (iii) before the laser was switched off. The red dashed circles indicate the positions of silica bead during one cycle of oscillation. Oscillation of silica beads on different plasmonic/PNIPAM hybrid substrates. (C) single Au NPs in AAO channels, (D) dimer Au NPs in AAO channels, and (E) trimer Au NPs in AAO channels. Insets are the schematics of corresponding hybrid plasmonic substrates. (F) Threshold laser power (*P*_th_) of silica bead oscillation and its moving distance on different hybrid plasmonic substrates.

Such thermal actuation can also be used to manipulate micro-object situated on the surface of this hybrid plasmonic substrate where an additional layer (~300 nm) of PNIPAM is postpolymerized to enhance its responsivity (Fig. 4A). The laser beam (641 nm, 8.6 mW) generates a local thermal gradient within the substrate via plasmonic heating effect, which triggers the phase transition of PNIPAM. This is evidenced by an expanding white region that appears within 0.1 s after the laser is switched on (Fig. 4B-i and ii). In the meanwhile, a silica bead that is 30 μm away from the beam center (the white dots circled by red dashed circle in Fig. 4B) starts to move radially toward the beam center with a distance of ~5 μm, stopping near the rim of the white region (Fig. 4B-iii). When the laser is switched off, the silica bead moves backward to its original position (Fig. 4B-iv to vi). The whole oscillation process can be reproduced for many cycles with alternative laser on and off (Movie S1). In addition, the oscillation frequency seems to increase as the irradiation power increases likely because of the enlarged photothermal effect (Movie S2). Likewise, the oscillation frequency increases with the number of Au NPs in the assemblies (Fig. 4C to E and Movies S3 and S4), as the photothermal conversion efficiency is higher for larger number of Au NPs in the channels (Fig. S4). Although this oscillation can also work on flat PNIPAM/Au NP films (~20 nm), it requires much higher power (~16 mW) to trigger such oscillatory movement (Fig. S5 and Movie S5). For AAO channels filled with Au NPs, the threshold power of oscillation decreases with increasing number of Au NPs (red line in Fig. 4F). The amplitude of silica bead oscillation is also larger on Au@PNIPAM/AAO substrates, which decreases with increasing number of Au NPs in the channels (blue bars in Fig. 4F). However, we find that the amplitude of oscillation is mainly determined by the thickness of PNIPAM films on top, which renders the oscillation amplitude up to 10 μm (Fig. S6 and Movie S6).

The force that drives the movement of silica toward the heated region is not from optical forces as it is too far away from effective range (~1 μm) of the laser beam. The white domain represents the region whose temperature is above the phase transition temperature of PNIPAM, which is much larger than the size of beam spot. In the meanwhile, the photothermal gradient (Fig. S7) induced from the Gaussian beam can generate thermophoretic forces [51], which, however, is very small (~10^−14^ N) in this system (Fig. S8); thus, it can also be ignored.

One possibility is the fact that the contraction of PNIPAM film under photothermal heating drives the movement of silica beads attached on the surface. The plasmonic heating effect in the laser-irradiated region leads to the phase change of PNIPAM film, resulting in a drastic volume shrinkage strain (up to *ε* ~ 0.7) [52]. The maximum stress induced by this shrinkage strain can be estimated by *σ* ~ *E* ∙ *ε*/3. By substituting the modulus of PNIPAM, i.e., *E* = 100 kPa [52], the maximum stress is calculated as *σ* ~ 23 kPa. Driven by such large contraction stress, the surface contraction displacement increases drastically from zero to the maximum, as the silica beads approach to the edge of the phase transition region, which then decays rapidly to zero again in the phase change region.

Another possible mechanism is the surface energy gradient-induced surface force, which is also incurred by the phase change of PNIPAM (Fig. S9) [52,53]. Such surface force can be calculated as (see the Supplementary Materials for the detailed modeling)

$$F_{∆\gamma }=-\;5C{\left(\frac{∆\gamma }{E^{*}}\right)}^{\frac{2}{3}}\nabla T\frac{\partial \left(∆\gamma \right)}{\partial T}$$ (1)

where ∆*γ* is the adhesion energy, $E^{*}=\frac{E}{1-\nu^{2}}$, and *E* and *v* are the modulus and Poisson’s ratio of the PNIPAM substrate, respectively. Here, $C=-\;\frac{2}{15}{\left(\frac{9}{8}\right)}^{\frac{2}{3}}\pi^{\frac{5}{3}}∙R^{\frac{4}{3}}$ is a constant related to the radius of the beads. Clearly, the surface force is a function of temperature, which changes at different positions. For the typical experiment in Fig. 4, the surface energy gradient-induced surface force can be 10^−8^ to 10^−7^ N (Fig. S9C).

The force that counteracts the surface energy gradient is the elastic force from the elastic modulus gradient of PNIPAM films. Such gradient is incurred by the increasing Young’s modulus (*E*) of PNIPAM film as a function of temperature (Fig. S9D) [52]. This elastic force can be calculated as (see the Supplementary Materials for details)

$$F_{e}=2C{\left(\frac{∆\gamma }{E^{*}}\right)}^{\frac{5}{3}}\nabla T\frac{\partial E^{*}}{\partial T}$$ (2)

It is noteworthy that the elastic force is much smaller than the force induced by the surface energy gradient in our experiments (Fig. S9D). Thus, for silica beads located near the outer edge of the phase transition region, the surface force can further pull the silica beads toward the beam center.

**Fig. 5.**
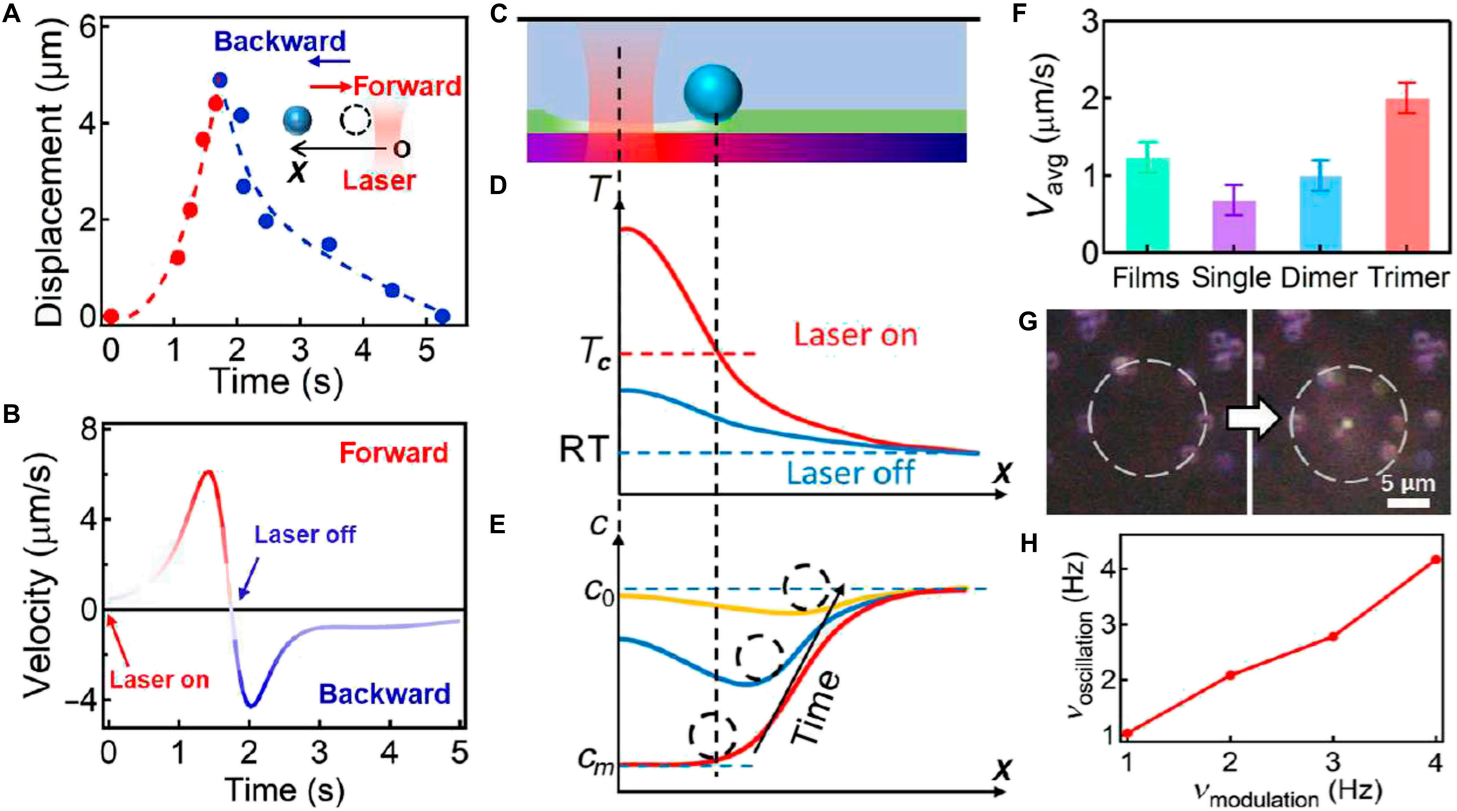
Mechanistic understanding of phototaxis movement of silica bead on hybrid plasmonic substrate. (A) Change of the silica bead’s position with time. Inset shows the schematic of the movement. (B) Velocity of forward and backward movements of silica bead at different time. Mechanism of the backward movement of silica bead after laser is switched off. (C) Schematic diagram of bead in equilibrium position after laser is switched on. (D) Schematic of temperature distribution in the PNIPAM substrate with laser switched on and off. *T*_c_ and R.T. are the phase transition temperature and the room temperature, respectively. (E) Schematic of water concentration distribution within the PNAPAM films after laser is switched off. The black dashed circles represent the position of silica bead, which is changing with time. *c*_0_ and *c_m_* are water contents of PNIPAM substrate when the temperature is R.T. and above *T*_c_, respectively. (F) Average speed of silica movement in one cycle of oscillation on different plasmonic hybrid substrates. The irradiation power is at the threshold power of the movement. (G) DF image of multiple silica beads before and after laser irradiation. The white dashed circles indicate the original positions of the silica beads, and the white spot at the center of the right panel indicates the irradiation beam spot. (H) Change of silica oscillation frequency with the modulation frequency of the laser.

As for single Au NPs in AAO channels with PNIPAM films on top, the silica bead takes ~5 s to accomplish a cycle (Fig. 5A) with variable speed (Fig. 5B and Movie S1). The driving force for the forward movement of the silica bead (inset of Fig. 5A) could be from the volume contraction of PNIPAM and the surface force incurred by surface energy gradient. The maximum speed can reach 6 μm/s but quickly drops to zero (red line in Fig. 5B), as it reaches the rim of the phase transition region where no surface energy gradient exists beyond this point (Fig. S9). When the laser is switched off, the white region fades away immediately within tens of milliseconds (Fig. 4B-iv), indicating that the temperature drops below the phase transition temperature of PNIPAM (Fig. 5C and D). This cooling dynamics is also supported by the simulation that the temperature around the silica bead (~20 μm away from the beam centre) decreases to the room temperature within several milliseconds (Fig. S10). As the inflation of PNIPAM with water occurs when the temperature drops below the phase transition temperature, the PNIPAM film quickly expands, which pushes the silica beads back to its original position (blue line in Fig. 5A). Such inflation process can be understood as the diffusional flow of water molecules into the PNIPAM matrix, which is proportional to its local concentration gradient [*c*_0_ − *c*(*x*)]. The concentration distribution of water molecules in the PNIPAM films will change with time as shown with the colored lines in Fig. 5E, which forms the local minimum of energy trap for the silica bead. With continuous water diffusion, the position of the local energy minimum shifts toward the cold region with decreased speed (black arrow in Fig. 5E), which is consistent with the experimental observation (blue line in Fig. 5B).

The average speed of SiO_2_ bead movement is also different on hybrid plasmonic substrate (Fig. 5F). For Au NP films fabricated by magnetron sputtering, the photothermal efficiency is much lower compared to Au NPs in AAO channels; thus, the silica beads show much smaller averaged speed but with much higher power threshold (Fig. 4F, Fig. S5, and Movie S5). With increasing number of Au NPs in the AAO channels, the average movement speed of silica beads also increases (Fig. 5F), which is attributed to the drastically increased photothermal efficiency of plasmonic NPs as the calculated local temperature increases with the number of Au NPs (Fig. S4). Thus, the Au@PNIPAM NPs/AAO hybrid plasmonic substrate shows superior performance on particle manipulation in terms of locomotive speed and range, which is capable of manipulating multiple particles at the same time. The actuation direction of these particles is always along the radial direction of the laser beam, which follows the exactly the gradient profiles of the temperature (Fig. 5G and Movie S7). This controlling capacity is much higher than optical trap which is normally one particle at a time. The oscillation frequency of the silica beads can also be programmed by modulating the laser beam, which robustly follows the modulation frequency (Fig. 5H and Movie S8). Higher modulation frequency is possible but limited by the spatial and temporal resolution of the optical microscope and camera. Nevertheless, this intriguing oscillation unveils the underlying correlations between photothermal effect and water diffusion, which induces the dynamic evolution of surface contraction/expansion displacement and surface forces.

## Discussion

In summary, we have fabricated a photothermal active hybrid plasmonic substrate via hydrodynamic self-assembly of Au@PNIPAM NPs with assistance of AAO template. Because of the coupling of photothermal effect and water diffusion in the PNIPAM films, the dynamic evolution of the surface contraction/expansion displacement of the PNIPAM overlayer and the surface forces drives the silica bead to show bistate actuation with programmed laser beam. The direction of the oscillation is along the radial direction of the laser beam, which can control multiple particles at the same time with interaction range up to 10 μm. The high photothermal efficiency of such hybrid plasmonic substrate requires CW laser irradiation with power of only a few milliwatts, which reduces the potential risk of material damage, making it an ideal method for biological applications. This type of smart plasmonic metasurfaces not only sheds light on physical mechanism of light-driven bistate colloidal actuation but also shows huge implications for biomimicking, cell manipulation, and mechanics.

## Materials and Methods

### Materials

Au NPs of 60 nm were obtained from BBI Solutions. Monodisperse silica microspheres (SiO_2_) with a diameter of 1 μm were purchased from Macklin. PNIPAM terminated with amine groups (PNIPAM-NH_2_, *M_n_* = 5,500) and 2-hydroxy-2-methylpropiophenone (HMPP) were purchased from Sigma-Aldrich. *N*-isopropylacrylamide (NIPAM) was obtained from Acros. Oxalic acid, sulfuric acid, chromic acid, and phosphoric acid were obtained from Sinopharm Chemical Reagent Co. Ltd. The well-polished aluminium foil (thickness, 0.2 mm) of high purity (99.999%) was supplied by Shenzhen Topmembranes Technology Co. Ltd. Deionized (DI) water was prepared from Millipore.

### Fabrication of AAO template with double opening

AAO templates with double open channels were prepared according to previous literature [54] .Briefly, Al foil was firstly oxidized in an oxalic acid solution (0.3 M) at a constant voltage of 40 V for 6 h, which was then removed by chemical etching in a mixture of phosphoric acid (5 wt%) and chromic oxide (1.8 wt%). The second anodic oxidation was performed at the same voltage for 2 min whose pores were enlarged using a phosphoric acid solution (5 wt%) at 30 °C for 10 min. The third anodization was conducted under a constant voltage of 20 V for 18 h in the sulfuric acid solution to generate a variable subpores. Finally, the Al substrate was removed in saturated copper chloride solution, and the barrier layer was removed in phosphoric acid to form open channels.

### Hydrodynamic self-assembly of AuNPs@PNIPAM NPs in the AAO templates

Au@PNIPAM core shell NPs were firstly formed by mixing 1 ml of AuNPs and 2 μl of PNIPAM (10 mg/ml), which were refluxed at 45 °C. The PNIPAM shell thickness is ~2 nm as confirmed by transmission electron microscopy (TEM) (JEM2010, FEF). The mixture was further concentrated with centrifugation (6,500 rpm for 15 min) and redispersed in 50 μl of water for vacuum-assisted assembly in the AAO channels. To realize the better control of the number of Au NPs in the channel, sequential filtration of monolayer Au NPs obtained by triphase transfer was performed [55,56]. Extra layer of PNIPAM was post-polymerized to fully fill the pores of AAO templates [57]. Specifically, 50 μl of mixture containing 47.5 μl of NIPAM (2 M) and 2.5 μl of HMPP (1 μl/ml) was dripped on the samples, followed by ultraviolet irradiation (302 nm) for 15 min [58]. Extra unbounded PNIPAM was removed by repeated washing with large amount of DI water and ethanol, leaving a ~300-nm overlayer on top.

### Tuning the coupled plasmons of AuNPs in AAO template and characterizations

The Au@PNIPAM NPs assembled in AAO templates were immersed in DI water with a coverslip for microscopic observation and spectral characterizations. The temperature of the solution was controlled with a Linkam heating stage with temperature between 20 and 45 °C. Scattering spectra were recorded through a DF microscope equipped with a 100× DF objective (BX53M, Olympus), which were confocally coupled to an optofiber spectrometer (QE65000, Ocean Optics). SEM images of the AuNPs in AAO templates were obtained at the accelerating voltage of 5 kV (Sigma, ZEISS).

### Dynamic oscillation of silica beads on thermal active plasmonic hybrid substrate

Silica beads of 1 μm were dropcasted on 4 types of hybrid plasmonic substrates (PNIPAM/Au NP films, PNIPAM/single Au NPs/AAO, PNIPAM/dimer Au NPs/AAO and PNIPAM/trimer Au NPs/AAO), which were then immersed in water with a coverslip for microscopic observation. A 641-nm CW laser with variable power was focused on the hybrid plasmonic substrate through a 100× DF objective to locally heat up the PNIPAM films, and the movement of silica beads was recorded at the frame rate of 8 to 10 fps with a 642 nm Notch filter (Semrock) placed in front of charge-coupled device camera (Infinity 3.0, Lumera). The laser beam was modulated with a square wave trigger from a function generator (33510B, Keysight) at varied frequency.

## Data Availability

The raw data of the plots shown in this paper can be available from the authors with reasonable request.

## Supplementary Materials

Supplementary 1 Fig. S1. Modeling of thermophoretic forces and surface forces. Fig. S2. TEM image of Au@PNIPAM NP. Fig. S3. SEM image of the AAO template. Fig. S4. Absorption spectra of single, dimer, and trimer Au NPs. Fig. S5. Calculated local temperature at the laser focal spot with increasing number of Au NPs in the AAO channels. Fig. S6. Movement of silica bead on flat Au NP films coated with PNIPAM. Fig. S7. Movement of silica bead on dimer Au NPs/AAO substrate coated with thick PNIPAM films. Fig. S8. Theoretical correlation of the surface energy, Young’s modulus with temperature, and different forces. Fig. S9. Calculated cooling dynmaics after laser is switched off.
