# Supplementary material for: Light-Programmed Bistate Colloidal Actuation Based on Photothermal Active Plasmonic Substrate
Source: Research (Wash D C). 2023 Jan 10;6:0020. doi: 10.34133/research.0020 (PMC10076013; doi:10.34133/research.0020)
Supplement: Supplementary 1 — Fig. S1. Modeling of thermophoretic forces and surface forces. Fig. S2. TEM image of Au@PNIPAM NP. Fig. S3. SEM image of the AAO template. Fig. S4. Absorption spectra of single, dimer, and trimer Au NPs. Fig. S5. Calculated local temperature at the laser focal spot with increasing number of Au NPs in the AAO channels. Fig. S6. Movement of silica bead on flat Au NP films coated with PNIPAM. Fig. S7. Movement of silica bead on dimer Au NPs/AAO substrate coated with thick PNIPAM films. Fig. S8. Theoretical correlation of the surface energy, Young’s modulus with temperature, and different forces. Fig. S9. Calculated cooling dynmaics after laser is switched off. [file research.0020.f1.pdf]

Supplementary Materials

Light-Programmed Bistate Colloidal Actuation Based on Photothermal Active Plasmonic Substrate

Fangfang Deng[1, †], Juntao Chen[2 ,†], Junxiang Xiang[2, †], Yong Li[1], Yan Qiao[3], Ze Liu[2,*] and Tao Ding[1,*]

1 Key Laboratory of Artificial Micro/Nano Structure of Ministry of Education, School of Physics and Technology, Wuhan University, Wuhan, 430072, China.

2 Department of Engineering Mechanics, School of Civil Engineering, Wuhan University, Wuhan, Hubei 430072, China

3 Beijing National Laboratory for Molecular Sciences (BNLMS), Laboratory of Polymer Physics and Chemistry, CAS Research/Education Center for Excellence in Molecular Sciences, Institute of Chemistry, Chinese Academy of Sciences, Beijing 100190, China

*Corresponding authors. E-mail: t.ding@whu.edu.cn (T. Ding); ze.liu@whu.edu.cn (Z. Liu).

[†]These auhtors contribute equally to this work.

This PDF contains

Modelling details

Fig.S1-S10

Legends of Video S1-S8

Thermophoretic forces

The thermophoretic forces induced by the photothermal effect can be calculated as

$$F_{th} = 6\pi R\mu D_T \nabla T$$

where R is the radius of silica bead, μ is the viscosity of the fluid, D_T is the thermal diffusion coefficient, $\nabla \mathrm{T}$ is the temperature gradient. The results are shown in Fig. S8.

Surface forces

Considering the adhesive contact between a rigid sphere (with a radius of R) and a semi-infinite elastic substrate, according to the JKR theory [1], the system energy under equilibrium is

$$U_{tot} = E^* \left(\frac{8}{15} \frac{a^5}{R^2} + \frac{\Delta\gamma\pi a^2}{E^*} - \frac{4}{3} \frac{a^3}{R} \sqrt{\frac{2\Delta\gamma\pi a}{E^*}} \right) \quad (1)$$

where a is the contact radius, $E^* = E/(1 - \nu^2)$ is the composite modulus, ν is the Poisson's ratio, $\Delta\gamma$ is the adhesion energy. In our experiments, no external load is applied, thus the contact radius of the sphere reads

$$a = \left(\frac{9}{8} \frac{\Delta\gamma\pi R^2}{E^*} \right)^{\frac{1}{3}} \quad (2)$$

Substituting eq. (2) into eq. (1) gives

$$U_{tot} = -\frac{2}{5} \left(\frac{9}{8} \right)^{\frac{2}{3}} \pi^{\frac{5}{3}} \cdot R^{\frac{4}{3}} \cdot E^{*-\frac{2}{3}} \cdot \Delta\gamma^{\frac{5}{3}} \quad (3)$$

Considering that both E^* and $\Delta\gamma$ are temperature dependent, and when laser is switched on or off, a temperature gradient generates, which induces surface forces acting on the silica beads from the gradient of modulus and adhesion energy, respectively. The total surface force can be calculated as

$$F = -\frac{\mathrm{d}U_{tot}}{\mathrm{d}x} = F_{\Delta\gamma} - F_e = C \left(\frac{\Delta\gamma}{E^*} \right)^{\frac{2}{3}} \frac{\mathrm{d}T}{\mathrm{d}x} \left[2 \frac{\Delta\gamma}{E^*} \frac{\partial E^*}{\partial T} - 5 \frac{\partial \Delta\gamma}{\partial T} \right] \quad (4)$$

where $C = -\frac{2}{15} \left(\frac{9}{8} \right)^{\frac{2}{3}} \pi^{\frac{5}{3}} \cdot R^{\frac{4}{3}}$ is a constant related to the bead radius. $F_{\Delta\gamma}$ and F_e are function of temperature/position, and the calculated surface forces associated with the typical experiment in Fig. 4b are plotted in Fig. S9.

Reference

[1] Popov, Valentin L. Contact mechanics and friction. Berlin: Springer Berlin Heidelberg, 2010.

Supplementary Figures

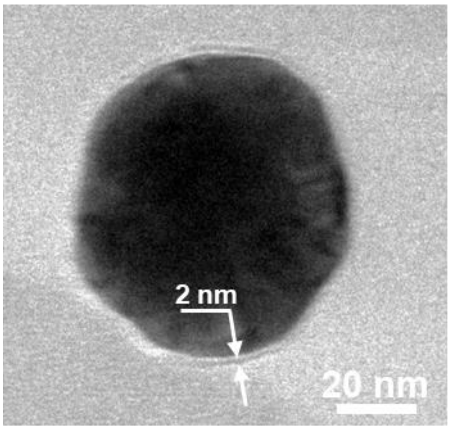

Fig. S1. TEM image of the Au@PNIPAM NP

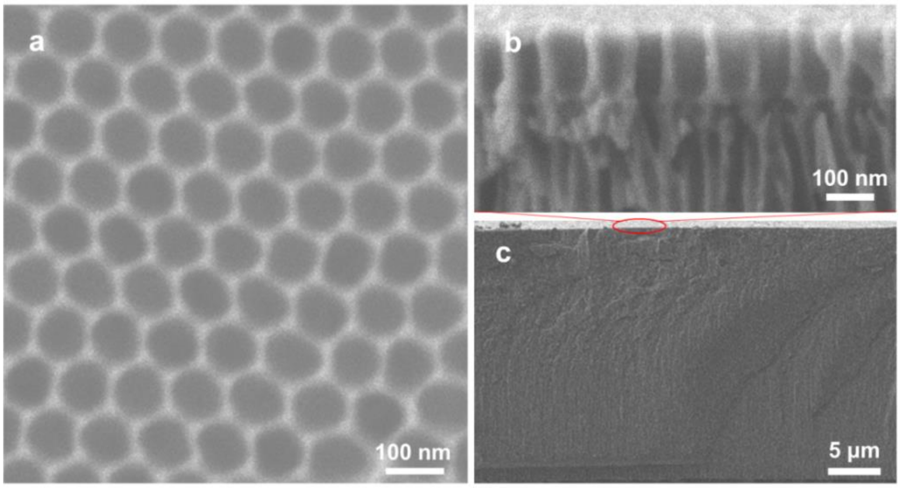

Fig. S2. SEM images of AAO template with double openings. (a) top view. (b, c) cross-section views. (b) is the enlarged image in the circled region of (c).

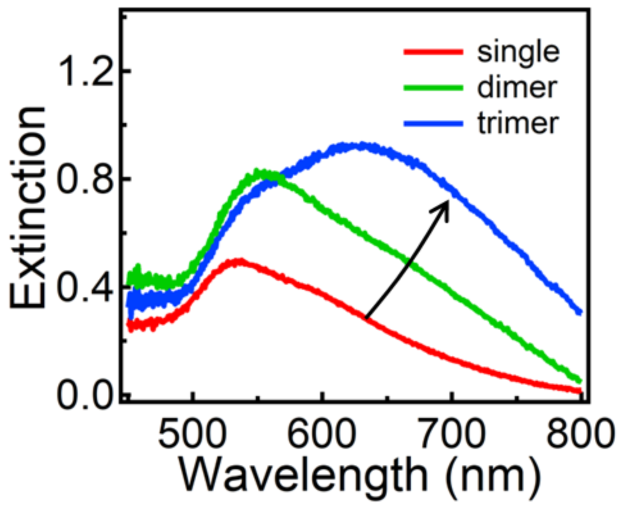

Fig. S3. Absorption spectra of single Au NPs, dimers and trimers in the AAO template.

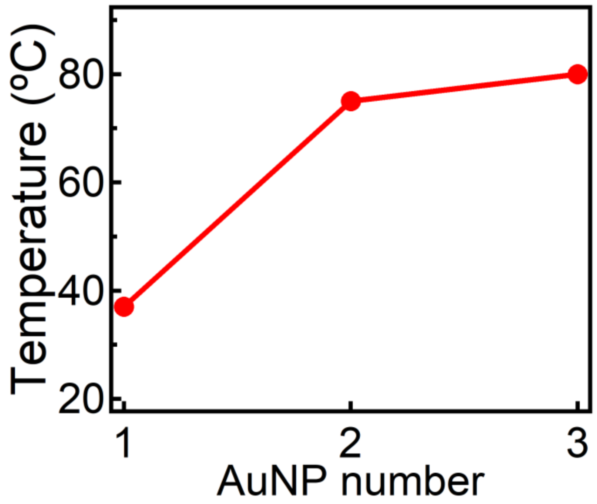

Fig. S4. Calculated local temperature of single Au NPs, dimers and trimers in AAO template. Irradiation power: 3 mW.

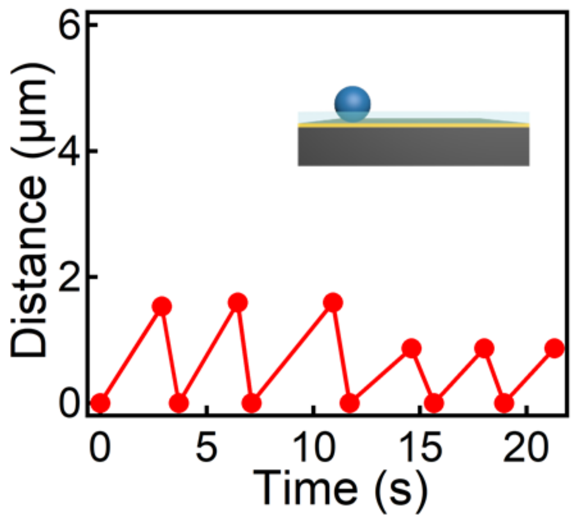

Fig. S5. Oscillation of silica beads on flat AuNP films/PNIPAM hybrid substrate. Irradiation power is 16 mW.

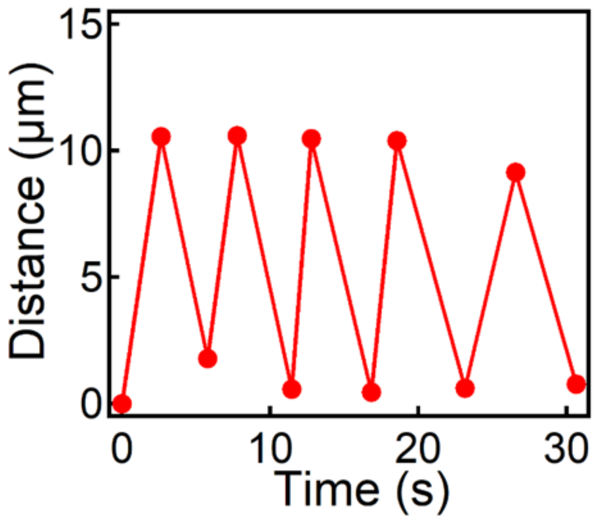

Fig. S6. Oscillation of silica beads on Au dimers/PNIPAM/AAO hybrid substrates with thicker PNIPAM films (~1 μm). Irradiation power is 3 mW.

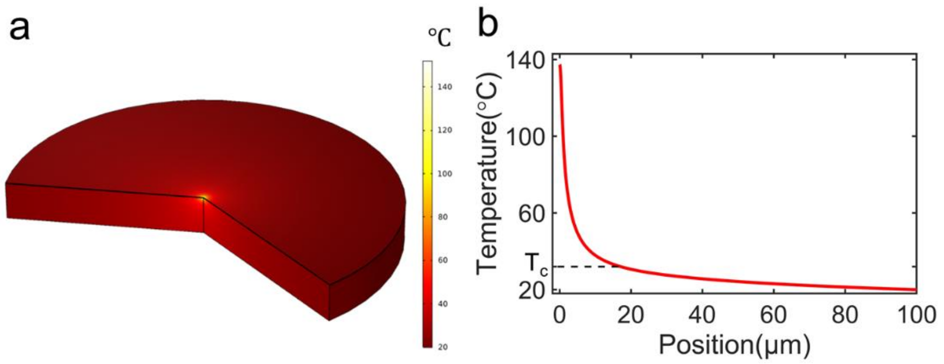

Fig. S7. Finite element analysis the heating process of a single Au NP@PNIPAM/AAO substrate when its centre is irradiated by a laser beam (the same condition as the experimental parameters in Fig. 4b). The substrate includes three layers. The top layer is PNIPAM (with thickness of 300 nm) with thermal conductivity of 0.16 W/m·K by approximation. The middle layer is the AuNPs/AAO composite layer (with thickness of 85 nm), and the bottom layer is AAO substrate (with thickness of ~20 μm). The thermal conductivity is 310 W/m·K for AuNPs/AAO composite and 9 W/m·K for AAO substrate. (a) Simulated steady state temperature profile under laser irradiation. (b) Temperature profile at the upper surface of PNIPAM films. T_c refers to the volume phase transition temperature of PNIPAM (~ 32 ℃).

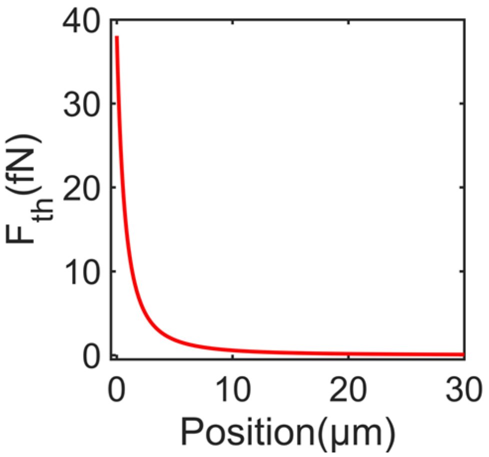

Fig. S8 Position dependent thermophoretic forces calculated based on the temperature distribution in Fig. S7b.

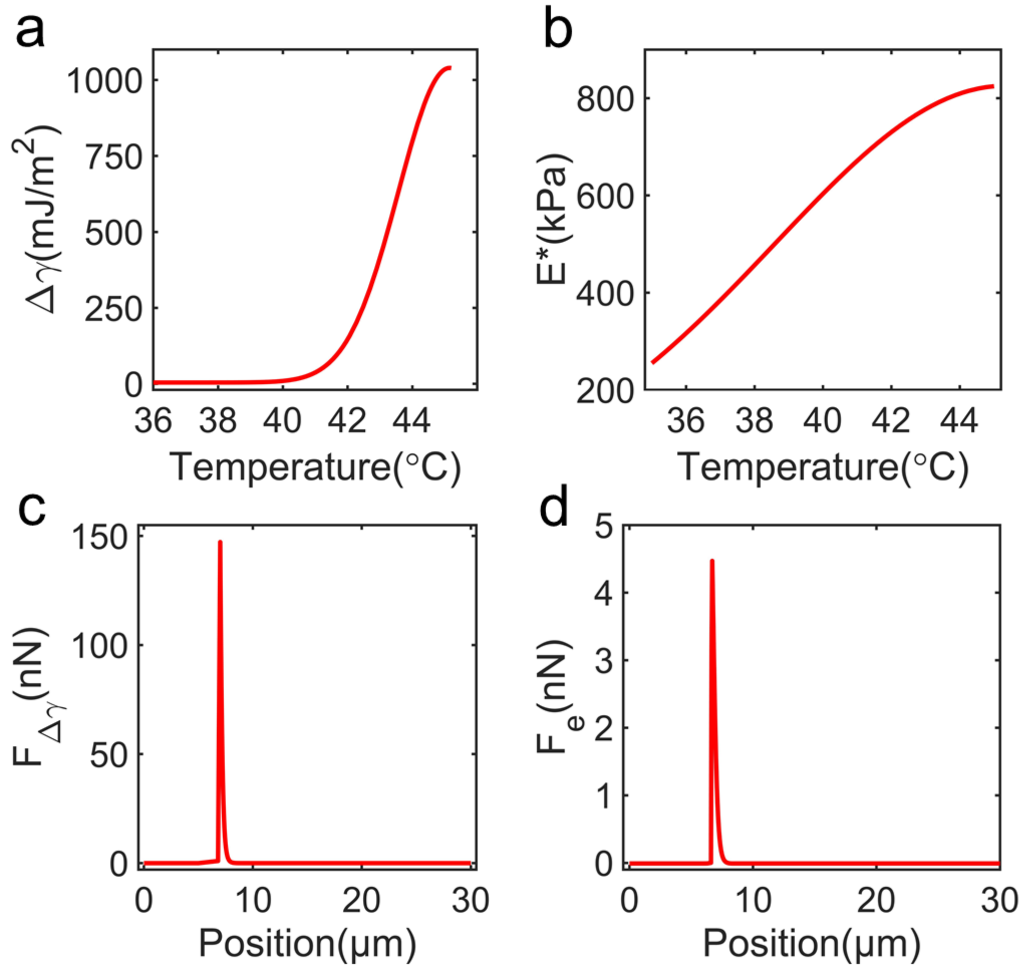

Fig. S9. Position dependent mechanical properties of the hybrid plasmonic substrate due to the laser induce temperature gradient (refers to Fig. S7b). Change of (a) adhesion energy and (b) Young's modulus of PNIPAM films with temperature. (c)-(d) Calculated surface forces of the silica bead at different positions of the PNIPAM surface, which are obtained based on the temperature gradient in Fig. S7b. The position is the distance measured from the silica bead to the beam centre.

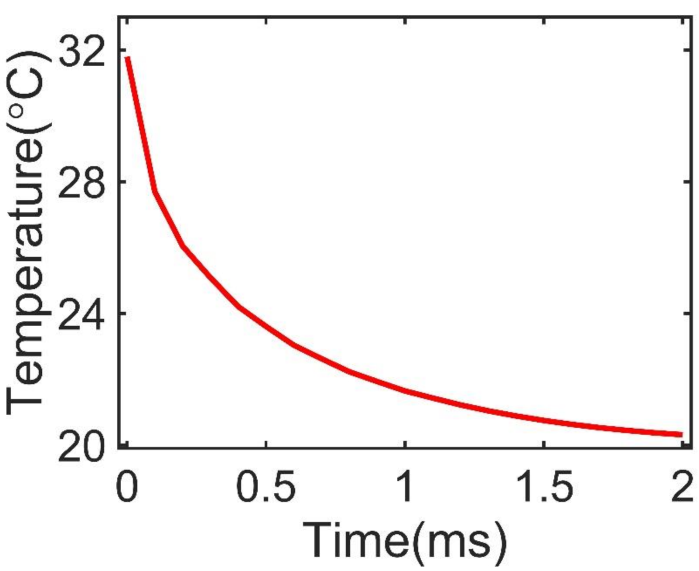

Fig. S10. Calculated temperature cooling with time at the position of the silica bead (the position with temperature T_c, Fig. 4b(iv)) after the laser is switched off. Refer to Fig. S7 for the simulation model.

Supplementary videos

Video S1: Oscillation of silica bead on PNIPAM/AAO template filled with single Au NPs with laser on and off. Laser power: 8.6 mW

Video S2: Oscillation of silica bead on PNIPAM/AAO template filled with single Au NPs with laser on and off. Laser power: 10.5 mW.

Video S3: Oscillation of silica bead on PNIPAM/AAO template filled with Au dimers with laser on and off. PNIPAM film thickness is ~300 nm. Laser power: 3 mW.

Video S4: Oscillation of silica bead on PNIPAM/AAO template filled with Au trimers with laser on and off. Laser power: 3 mW

Video S5: Oscillation of silica bead on PNIPAM/AuNP films with laser on and off. Laser power: 16 mW

Video S6: Oscillation of silica bead on PNIPAM/AAO template filled with Au dimers with laser on and off. PNIPAM film thickness is ~1 μm. Laser power: 3 mW.

Video S7: Radial oscillation of multiple silica beads on PNIPAM/AuNP films.

Video S8: Fast oscillation of silica beads with repeated laser on and off.
